# Surgical excision methods for skin cancer involving the nail unit: A systematic review

**DOI:** 10.1002/cesm.12026

**Published:** 2023-10-16

**Authors:** Claire M. Hardie, Ryckie G. Wade, Justin C. R. Wormald, Brian Stafford, Faye Elliott, Julia Newton‐Bishop, Donald Dewar

**Affiliations:** ^1^ Leeds Institute of Medical Research University of Leeds Leeds UK; ^2^ Department of Plastic and Reconstructive Surgery Leeds Teaching Hospitals NHS Trust Leeds UK; ^3^ Nuffield Department of Orthopaedics, Rheumatology and Musculoskeletal Sciences (NDORMS) Oxford UK; ^4^ World Health Organization/Consumers Health Forum/Health Consumers' Council of WA Perth Australia

## Abstract

**Introduction:**

Skin cancer affecting the nail unit is rare but is associated with morbidity, and melanoma has a high mortality rate. The principal treatment is surgical excision and methods can be classified into digit‐sparing surgery or amputation. Digit‐sparing surgery (wide excision or Mohs surgery) may be safe and effective for malignancies involving the nail unit in comparison to amputation if there is not bony invasion. The objective was to assess the efficacy and safety of different methods of surgical excision for skin cancer involving the nail unit.

**Methods:**

Prospective comparative studies (randomized controlled studies, non‐randomized controlled studies and prospective observational studies) of surgical excision for skin cancer of the nail unit in all participants were eligible for inclusion. We searched electronic databases, trials registers and conference abstracts. We checked the reference lists of included studies and related systematic reviews for further references to relevant studies, and we contacted experts to enquire if they were aware of any additional relevant trials. We used standard methodological procedures expected by Cochrane. The primary outcomes were overall survival, disease free survival and adverse events/outcomes at 30 days. The secondary outcomes were quality of life outcomes. We planned to use GRADE to assess the quality of the evidence for each outcome.

**Results:**

We did not identify any studies that met the inclusion criteria for this review. We have been unable to assess our outcomes of overall survival, disease free survival, adverse events/effects and quality of life.

**Conclusions:**

As we have not identified any studies for inclusion, we are unable to assess the efficacy and safety of different methods of surgical excision for skin cancer involving the nail unit. We suggest that comprehensive cancer registry analysis is required in this field to obtain meaningful data.

## Plain language summary

Surgical excision methods for skin cancer involving the nail unit.

Review Question

What is the safest and most effective method of surgical removal of skin cancers involving the nail?

Background

Skin cancer affecting the skin under or around the nail (nail unit) is rare but can cause significant problems for the patient and can have a high death rate. Fingers and toes can both be affected, and can be referred to as a “digit.” The main treatment for these conditions is complete removal with surgery which can be grouped into digit‐sparing surgery or amputation. Digit‐sparing surgery may be safe and effective compared to amputation with better function and appearance, but there is a mix of evidence on the risk of the cancer coming back.

Study Characteristics

We searched electronic databases for journal papers, references from review papers, conference abstracts, and asked experts if they were aware of any ongoing studies. We did not find any studies that met our inclusion criteria.

Key Results

Unfortunately, we did not find any good quality evidence to include in this review as the published work available was done as reviews of patient notes (retrospective), rather than as planned (prospective) studies.

Author's Conclusions

We cannot give any firm conclusions on the management of skin cancer of the nail unit. Large cancer registry studies are needed to give evidence on the best management of the condition.

## 1 INTRODUCTION

Skin cancer arising on the digits of the hands or feet is rare, and occasionally affects the nail unit. The nail unit comprises the nail plate, which arises from the nail bed and is surrounded on all sides by supporting soft tissues and their neurovasculature. These structures are adherent to the periosteum of the distal phalanx and closely related to the insertion of the terminal extensor tendon [1, 2]. Squamous cell carcinoma (SCC) is the most common malignant tumor involving the nail unit, although it is rare and the exact incidence is unclear [3]. Tumors involving the nail have a higher rate of recurrence than SCCs in other sites [4], however death from metastatic SCC originating on a digit is rare and only a few cases have been reported [3]. Cutaneous melanoma involving the nail unit is a rare variant accounting for approximately 1%–3% cases of melanoma in the white population [5, 6]. Five‐year survival rates with this condition vary from 16% to 80% [7], with more recent studies demonstrating a median overall survival of 40–55 months [8, 9]. Basal cell carcinoma (BCC) of the nail unit is the most rare, with only case reports available in the literature [10]. Metastasis of any BCC is extremely rare [11] and rates of recurrence for BCCs of the nail unit are not reported. Staging of these cancers is done according to the eighth edition of the American Joint Committee on Cancer (AJCC) system [12]. The principal treatment for cutaneous SCC, melanoma and BCC is surgical excision. However, several different methods of surgery are utilized for skin cancer involving the nail unit. The options can be challenging to resolve for patients and surgeons, due to the desire to preserve the length of the digit and the conflicting need for wide excision in the form of an amputation [13]. Overall, these options can be simplified into surgery that preserves the digit or amputation.

### 1.1 Digit‐sparing surgery

For malignancies involving the nail unit, surgeons may elect to remove the tumor and subcutaneous tissues but spare the skeleton and major structures of the digit (tendons, nerves, vessels, etc.) which is called wide excision. The recommended margins for SCC are 4 mm for low‐risk tumors and 6 mm for high‐risk tumors or tumor with histological thickness of more than 6 mm [14]. For cutaneous melanoma the recommended peripheral excision margin depends on the Breslow thickness of the tumor; in the UK, the suggested clinical margins range from 1 to 3 cm depending on tumor thickness [15]. However, excision margins remain strongly debated and most of the studies concerning peripheral margins excluded tumors on the extremities [16]. The recommended margins for BCC are 4 mm for low‐risk tumors and up to 15 mm for high‐risk tumors [17]. Mohs micrographic surgery is a margin‐controlled excision technique which uses intraoperative stepwise histological examination of the margins by the operating surgeon [18, 19]. When digit‐sparing surgery is performed (whether by conventional wide excision or micrographic surgical means) the aim is to enable reconstruction by means of skin graft(s) or flap(s) to retain a useful digit.

### 1.2 Amputation

When preservation of the digit is not desired or possible, amputation may be performed. Indeed, such surgery is recommended by many groups for both primary SCC [20] and melanoma involving the nail unit [21, 22, 23]. Shortening digits may confer cosmetic and functional impairment but offers improved chances for complete excision [24]. The digit can be amputated at various levels (typically disarticulation at the distal or proximal interphalangeal joints) depending on the desired margin of clearance, expected residual function and the patient's wishes.

### 1.3 Rationale

Digit‐sparing surgery may be effective in treating malignancies involving the nail unit without the associated morbidity of amputation. There is conflicting evidence regarding the risk of recurrence for both SCC and melanoma when digit‐sparing surgery is used, compared to amputation [20, 25]. The concern with digit‐sparing surgery over amputation is that the narrower margins used may be associated with increased risk of recurrence [26]. Digit‐sparing surgery also significantly reduces the deep margin due to the paucity of soft tissue between the nail unit and distal phalanx [27]. Skin cancer of the nail unit is associated with significant morbidity and melanoma has a high mortality rate. There is a diversity in practice in the management of these cancers involving the nail unit and there are increasing rates of digit‐sparing surgery reported in the literature. However, any difference in outcomes between digit‐sparing surgery and amputation are not clear, nor is it clear how these interventions affect patients' quality of life. These are important considerations for patients and clinicians when deciding on treatment for these conditions, and by undertaking this review we wished to provide a comprehensive summary of the treatment options and associated outcomes.

### 1.4 Objectives

To assess the efficacy and safety of different methods of surgical excision for skin cancer involving the nail unit.

## 2 MATERIALS AND METHODS

For complete methods please see the study protocol [28].

### 2.1 Eligibility criteria

We were aware of the limited evidence from randomized controlled trials (RCTs) in this clinical area, hence we considered more than one study design. We planned to carry out all assessment and reporting separately for each study design. The following study types were eligible for consideration, without restriction on language or publication status.

- RCTs, including quasi‐ and cluster‐RCTs. Cross‐over trials were excluded as this design is inappropriate for the clinical condition under examination.
- Nonrandomised controlled studies of surgical management of nail unit cancers.
- Longitudinal observational studies directly comparing digit‐sparing surgery to amputation. These include observational studies of prospective cohort or those of nested case‐control design with an appropriate comparator group. Case series and case reports were excluded.

We included participants of all ages and ethnic groups, with histologically proven primary cutaneous SCC, melanoma or BCC of the finger, toe or thumb, which is associated with the nail unit. Tumors of all histological thicknesses (AJCC stage 0, I, II, or IIIa; [12]) would be included, and those without documented histological thickness would be considered. Tumors without a defined histological thickness are common in the nail unit as the initial incision biopsy can disrupt measurement.

The interventions of interest for nail tumors were wide local excision, Mohs surgery and amputation. Studies comparing any of the three interventions were considered.

**Primary outcomes**

1. Overall survival, defined as from date of diagnosis to date of any cause death at 5 years (or the closest time point to 5 years).
2. Progression free survival, defined as from date of diagnosis to date of any disease progression or any cause death at 5 years (or the closest time point to 5 years).
3. Adverse events/outcomes, to include any wound problem within 30 postoperative days (e.g., infection, wound breakdown, need for reoperation).

**Secondary outcomes**

1. Quality of life (e.g., measured using the EuroQol EQ‐5D [29], Medical Outcomes Study Short‐Form Health Survey (SF‐36) [30], Sickness Impact Profile (SIP) [31] or Quality of Well‐Being (QWB) scale [32]). Quality‐of‐life outcomes would be assessed at 6 months (or the closest time point to 6 months).

### 2.2 Information sources and search strategy

We aimed to identify all relevant studies regardless of language or publication status (published, unpublished, in press, or in progress).

The Cochrane Skin Information Specialist (Liz Doney) searched the following databases up to 10 May 2022 using strategies based on the draft strategy for MEDLINE in our published protocol [33].

- The Cochrane Skin Specialised Register 2022 [34].
- The Cochrane Central Register of Controlled Trials (CENTRAL); 2022, Issue 4, in the Cochrane Library.
- MEDLINE via Ovid (from 1946 onwards).
- Embase via Ovid (from 1974 onwards).

Full search strategies are available in Supporting Information: File 1.

We (C. M. H. and R. G. W.) searched the following trials registers/portals using the terms: (nail or subungual) and (cancer or tumo/ur or neoplasm or melanoma or carcinoma) up to May 10, 2022.

- The World Health Organization International Clinical Trials Registry Platform (ICTRP) (apps.who.int/trialsearch/).
- ClinicalTrials.gov (www.clinicaltrials.gov).

The references of relevant reviews [7, 35, 36, 37] were searched for further references to relevant studies.

We contacted experts in the management of skin cancer on May 2022 to enquire if they are aware of additional relevant trials (see Table 1).

**Table 1:** Experts contacted regarding any additional relevant studies.

Role of expert	Response
Skin cancer specialist professor of plastic and reconstructive surgery Manchester, UK	Not aware of any further studies
Skin cancer specialist consultant plastic and reconstructive surgeon Hull, UK	Not aware of any further studies
Skin cancer specialist consultant plastic and reconstructive surgeon London, UK	Not aware of any further studies

We searched Web of Science's Conference Proceedings Citation Index up to May 10, 2022. Conference abstracts from the following meetings were handsearched if they were not already included in the Cochrane Skin Specialised Register or Embase.

- Journal of Plastic, Reconstructive and Aesthetic Surgery (European Society of Plastic and Reconstructive Surgery conference).
- British Association of Plastic and Reconstructive Surgery abstract archives.
- American Society of Plastic Surgery abstract archives (Plastic Surgery: The Meeting).
- Canadian Journal of Plastic Surgery (Canadian Society of Plastic Surgery Annual Meeting).

### 2.3 Selection process

The titles and abstracts of deduplicated references were downloaded to Covidence systematic review software [38], which was used for the primary screening. All titles and abstracts were screened by two independent review authors (C. M. H. and R. G. W.). Full texts were downloaded for all potentially relevant studies and two independent review authors determined final eligibility (C. M. H. and R. W.). Any disagreements were resolved by consensus or with input from a third review author (J. C. R. W.). Serial publications from the same data set were excluded.

### 2.4 Data collection process

We planned to collect and analyze data pertaining to SCC, melanoma and BCC separately as they are distinct clinical entities. We planned to collect these items on a piloted customized pro forma. Two independent review authors (C. M. H. and R. G. W.) would extract the data which would then be checked by a third author (J. C. R. W.) and entered into Cochrane's review‐writing software, RevMan Web [39]. Authors would not be blinded to the study authors, institution or journal.

### 2.5 Data items

We planned to collect the following information and enter it into a “Characteristics of included studies” table: study design and methods; participant characteristics (age and sex); study setting; tumor characteristics (type of malignancy, site, size depth, invasion, and ulceration); participant immunosuppression; intervention (wide local excision, Mohs surgery or amputation, including details of margins obtained/level of amputation); duration and timing of follow‐up; details of the primary outcomes and method of analysis.

### 2.6 Study risk of bias assessment

We planned to assess the risk of bias of included studies using the guidance from appropriate sections of the *Cochrane Handbook for Systematic Reviews of Interventions* [40]. We planned to assess risk of bias for the results included in the “Summary of findings” tables.

For randomized studies, we planned to use version 2 of the “Risk of bias” tool (RoB 2) [41], which provides algorithms and signaling questions to assess risk of bias. The effect of interest was assignment to treatment. The domains in the RoB 2 tool are: bias arising from the randomization process; bias due to deviations from intended intervention; bias due to missing outcome data; bias in measurement of the outcome; and bias in selection of the reported results [40]. We planned to answer a number of signaling questions resulting in the tool algorithm classifying each domain as “high risk of bias,” “low risk of bias,” or “some concerns.” The tool algorithm would also decide whether the overall risk of bias is “high risk,” “low risk,” or “some concerns.” To undertake these assessments we planned to use the RoB 2 Excel Tool [42]. We did not anticipate finding any cluster‐RCTs, but if this had been the case then we planned to use RoB 2 but with a domain specific to cluster‐RCTs from the archived version of the tool added (Domain 1b—”Bias arising from the timing of identification and recruitment of participants”) [43]. We made use of the guidance in the *Cochrane Handbook for Systematic Reviews of Interventions* [40].

For non‐randomized studies we planned to use the Risk Of Bias In Non‐randomized Studies (ROBINS‐I) tool [44]. We planned to examine all the domains of ROBINS‐I, which are: bias due to confounding; bias in participant selection; bias in classification of interventions; bias due to deviation from intended interventions; bias due to missing data; bias in measurement of outcomes and bias in selection of the reported result [44]. We planned to classify the study as: no information on which to make a judgment, or low, moderate, high, or critical risk of bias. If any studies reach critical risk of bias in any domain we planned to not continue with the assessment as those studies will be excluded from the main effects analysis, according to ROBINS‐I guidance.

Covariates can be an issue in nonrandomised studies and can occur when variables (or factors) may be involved in predicting the initial intervention received [44]. In this study we planned to consider tumor stage/Breslow thickness to be covariates due to possible preferences of treating patients with early disease more conservatively. The healthcare systems participants are treated in will also be considered as covariates due to potential differences in stage of diagnosis and local treatment preferences. These were defined following discussion with experienced clinicians and review of the literature. There are no cointerventions of interest to consider here.

We planned for assessment of risk of bias to be performed by two independent review authors (C. M. H. and R. G. W.) with any disagreements resolved by consensus or with input from a third author (J. C. R. W.). To summarize the assessment, we planned to present the risk of bias in a “Risk of bias” graph and provide a narrative summary. Outcomes may have been measured at different time points between the studies which we planned to discuss.

### 2.7 Effect measures

We planned to report hazard ratios (HRs) and 95% confidence intervals (CIs) for the outcomes of disease‐specific survival and overall survival, depending on the quality of data available. We planned that if either adjusted or unadjusted HRs are not available, we would attempt to extract any available data for the estimation of odds ratios (ORs) or risk ratios (RRs) and 95% CIs. For continuous outcomes or outcomes measured in scales (such as pain) we planned to express the mean difference (MD) and standard deviation. If different scales are reported, then we planned to calculate the standardized mean difference (SMD), where appropriate. For analysis of adverse events we planned to use RRs and 95% CIs.

Following advice from the *Cochrane Handbook for Systematic Reviews of Interventions* [40], we planned to consider combining groups for single pair‐wise comparisons, for example, digit‐sparing surgery compared to amputation. For participants who initially had digit‐sparing surgery then subsequently went on to have amputation for any reason, we planned to consider the primary surgery as the intervention and the amputation as an adverse outcome. We found no cluster‐RCTs but if they were included the appropriate adjustments would have be made if the required information was available, according to the *Cochrane Handbook for Systematic Reviews of Interventions* [40].

We anticipated that there would be be heterogeneity between the studies due to the variation between tumors and the use of varying surgical techniques. We planned to describe potential sources of clinical heterogeneity and downgrade the certainty of evidence according to GRADE criteria [45]. If pooling of studies was feasible, we planned to visually inspect between‐study heterogeneity on a forest plot for outlying studies and variability of estimated effects between studies, alongside a *χ* ^2^ statistic with CIs. We planned to use a a low *p* value (i.e., less than 0.10) as significant evidence of heterogeneity [40]. We all planned to assess statistical heterogeneity using the *I* ^2^ statistic. The heterogeneity would have been interpreted as potentially unimportant if the *I* ^2^ statistic is 0% to 40%; likely to be moderate if the value is from 50% to 80%; and substantial if the value is above 80%. If the *I* ^2^ statistic was high (over 80%) we planned to explore this further and consider not pooling the data, after checking the direction of the effect [40].

### 2.8 Synthesis measures

We planned to carry out any data synthesis and report this separately for all considered study designs. Data pertaining to SCC, melanoma and BCC would be analyzed separately as they are distinct clinical entities. We planned for this to be done for each of the four outcomes comparing digit‐sparing surgery (wide local excision or Mohs surgery) to amputation (total 12 comparisons).

Before any pooling of data, we planned to assess the bias of included studies and the effects only pooled if participants of included studies are clinically homogeneous. We planned to perform meta‐analysis for studies with comparable participants and methodology that are not at critical risk of bias [41], using RevMan Web [39]. We planned to only pool similar effect measures where sufficient data are available.

For time‐to‐event outcomes we planned to use either generic inverse variance modeling using hazard ratios [46] or comparison of dichotomous outcomes (Maentel‐Hanzel). We would have preferrred the adjusted HRs if there is similarity between the studies in their chosen adjusting variables, otherwise the unadjusted HRs will be used.

We expected the outcome data to be presented in various formats, from simple proportions through to time‐to‐event data; we planned to to recover as much data as possible, as explained above. We expected the quality‐of‐life data to be reported as a scalar value; if the same scales are used then we planned to present mean differences (MDs) between groups. If different quality‐of‐life tools are reported, then we planned to present between‐group differences using the standardized mean difference (SMD). We anticipated clinical heterogeneity and planned to use random‐effects models in all analyses. We planned that if there was substantial heterogeneity which could not be explained using subgroup or sensitivity analyses (or both), or the studies are at critical risk of bias, then a meta‐analysis would not be performed and instead the original data would be presented in tables.

### 2.9 Assessment of reporting biases

As no studies were included a funnel plot was not used to assess publication bias [47].

### 2.10 Certainty assessment

The GRADE approach was planned to be used to assess the certainty of evidence for each outcome. GRADE includes the assessment of five factors: study limitations (risk of bias), inconsistency of results, indirectness of evidence, imprecision, and publication bias [48]. Each outcome can be downgraded by one or two levels for each domain, and the overall certainty would be classed as either high, moderate, low or very low. Two review authors (C. H. and R. G. W.) were planned to perform the GRADE assessments independently, with any disagreements resolved via discussion or input from a third review author (J. C. R. W.). We planned to use GRADEpro GDT to create the summary of findings tables [49].

## 3 RESULTS

The searches of the databases (see Supporting Information: File 1) retrieved 3370 records with duplicates removed automatically. Our searches of the trials registers identified no further studies. From reference lists two potentially eligible further studies were identified. Therefore, we screened a total of 3372 records.

We excluded 3335 records based on titles and abstracts. We obtained the full text of the remaining 37 records; one was found to be a duplicate. We excluded all 36 studies.

**Figure 1:**
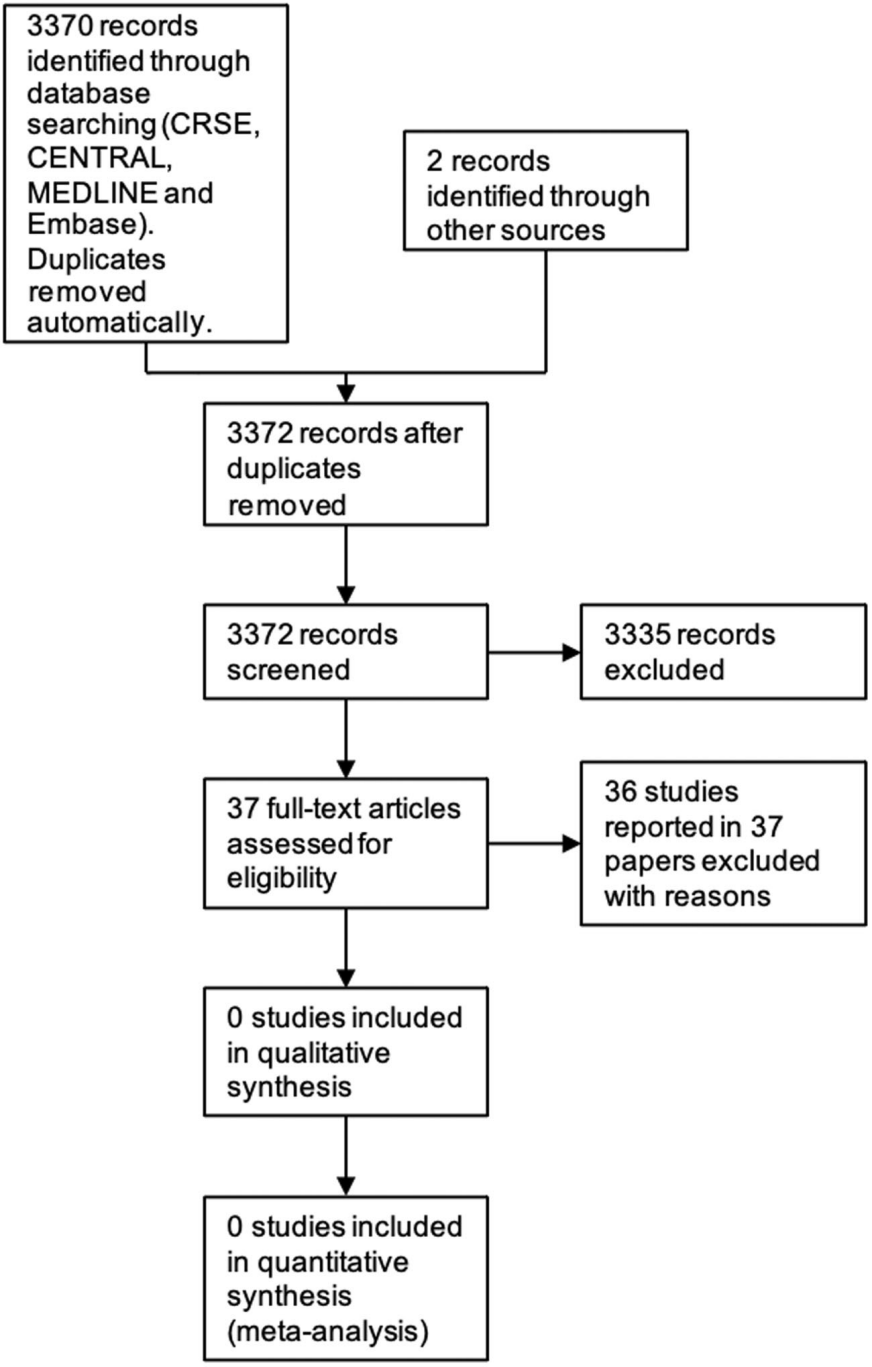
Study flow diagram.

For a further description of our screening process, see the study flow diagram (Figure 1).

We did not find any studies suitable that met the eligibility criteria.

We excluded 36 studies. The reason for exclusion is listed for each study in Characteristics of excluded studies (Supporting Information: File 2). Twenty‐five studies were excluded due to the study design (most frequently retrospective observational design). Seven studies evaluated the wrong intervention(s). Four studies were evaluating the wrong patient population.

We have not identified any ongoing studies that would be suitable for future inclusion in this review.

## 4 DISCUSSION

We did not include any studies in the current review. Due to lack of quality data we were unable to assess the efficacy and safety of different methods of surgical excision for skin cancer involving the nail unit.

We used a broad search strategy to incorporate multiple types of studies, to reduce the chance of missing relevant studies. Lower methodological quality studies were not included to reduce any bias in the findings, however this has limited the potential to discuss the results of these studies. Subjective judgment has been used in the assessment of their methodology, but studies have been reviewed by two authors independently with any disagreements resolved by discussion or involving a third author.

Cochran et al. [7] conducted a systematic review of treatment of nail unit melanoma. However, this review included retrospective studies, and studies without a comparator group both of which were not included in this review. The authors identified that both amputation and digit‐sparing surgery (wide local excision and Mohs surgery) were used in the treatment of nail unit melanoma, and that digit‐sparing excision appeared to have similar outcomes for melanoma in situ. Further conclusions were not drawn due to the limited evidence.

Jo et al. [36] reviewed local recurrence rates in digit‐sparing surgery in comparison to amputation for nail unit melanoma (in situ or minimally invasive) and performed a meta‐analysis. Five studies were included with a total of 109 patients, all of which were of retrospective design. Their meta‐analysis did not identify a significant difference between either treatment in terms of local recurrence, but was based upon a small amount of low‐quality evidence. The authors recommend digit‐sparing surgery in the first instance for in situ and minimally invasive cases of nail unit melanoma, to avoid severe functional deficit following amputation of the digit. The functional deficit experienced by patients following either type of surgery has not been assessed in this review.

Lieberherr et al. [35] undertook a systematic review and meta‐analysis of the treatment of nail unit melanoma with either amputation or wide local excision. Thirty articles of retrospective design were included. Meta‐analysis was undertaken to evaluate overall survival and progression‐free survival in this patient cohort, without comparing the two interventions. The authors believe that wide local excision should be aimed for in noninvasive cases, but did not draw further conclusions.

These systematic reviews on nail unit melanoma had broader eligibility criteria for included studies, including studies with retrospective and non‐comparative design. Although digit‐sparing surgery may be safe and effective for in situ or early stage melanoma this review has not found any conclusive evidence. No systematic reviews for SCC or BCC of the nail unit were identified.

In the excluded data, there was reported evidence for the surgical management of melanoma of the nail unit. This data was excluded from analysis due to either being retrospective in study design, or as the study reported on the outcomes of only a single intervention without comparator.

The J‐NAIL study [50] is an ongoing prospective single‐arm study evaluating the safety and efficacy of nonamputative digit sparing surgery for nail unit melanoma (stage I, II or III without evidence of tumor invasion to the distal phalanx). This is a single‐arm study but there is a planned comparison with outcomes from the Japanese Melanoma Study [51], which used amputation as the standard of care. The study plans to recruit for 5 years and then follow up for 5 years. This study may provide useful prospective evidence on the surgical management of nail unit melanoma. We identified no ongoing studies that provide evidence for the management of SCC or BCC of the nail unit.

In the excluded studies, other studies of some interest were those comparing digit‐sparing surgery and amputation.

Chakera et al. [52], Cohen et al. [24], Dika et al. [53] and Moehrle et al. [54] conducted retrospective reviews of their cancer registries to compare amputation to digit‐preserving surgery for patients with nail unit melanoma. The studies contained 103, 49, 39, and 62 patients, respectively. Chakera et al. managed three patients with in situ melanoma with wide excision and the rest with amputation, concluding that amputation at the DIPJ appeared safe. Based upon small, retrospective studies Dika et al. and Moehrle et al. did not identify any significant difference in survival or recurrence for patients treated with either amputation or digit‐sparing surgery. Cohen et al. reported in several cases the need for subsequent amputation for either recurrence or positive margins for patients managed initially with wide local excision.

Montagner et al. [55] undertook a retrospective review of 21 patients with nail unit melanoma with a mix of in situ and invasive disease. Amputation was performed in nine cases and 12 were managed with wide local excision. Two patients undergoing wide local excision and two who had amputation had recurrence of the melanoma with the 2‐year follow‐up period. The authors concluded that the surgical approach does not affect the prognosis of nail melanoma based on these observations. Park et al. [56] undertook a retrospective review of the management of 18 cases of nail unit melanoma in situ. Two patients underwent amputation at the DIPJ and 16 had wide local excision. No patients experienced recurrence of the melanoma, however the follow up period was short at only 2 months for some patients. Goettmann et al. (2018) undertook a retrospective cohort study of patients with in situ nail unit melanoma. Sixty‐three cases were identified, 56 patients had wide excision, and seven had amputation at the DIPJ. During a mean 10 year follow up two patients had in situ recurrences following wide excision. The authors conclude that digit‐sparing surgery should be the primary choice of treatment for in situ melanoma of the nail unit.

These excluded studies were of lower methodological quality so could not be included in this review. However, this is the level of evidence clinicians currently have to base practice upon.

## 5 CONCLUSIONS

Unfortunately, we did not identify any studies that met the inclusion criteria for this review so it has not been possible to meet our objective to assess the efficacy and safety of different methods of surgical excision for skin cancer involving the nail unit.

There is no suitable evidence available for this review on the efficacy and safety of methods of surgical excision for skin cancer involving the nail unit for either SCC, melanoma or BCC. Large randomized controlled studies would be ideal but may be difficult to achieve for these rare conditions. Multicentre prospective comparative studies of adults with skin cancer of the nail unit could provide useful data. However, both of these study methodologies could potentially lead to additional risks to patients due to the study of digit amputation, which is likely to be excessive in many cases.

We advocate for compilation of existing evidence into cancer registries in a manner which allows for meaningful causal analysis of retrospective data. This would allow the use of information that is already available to reach useful conclusions on this subject, ensuring that patients are not subjected to likely unnecessary digit amputation. We suggest within England that using NHS Digital cancer registry data with cases ascertained with data from Hospital Episode Statistics would provide a acceptable data set. If patients with a selected diagnosis of nail unit melanoma were included over a 20 year retrospective period this would allow a 10‐year survival follow up. Data on demographics, diagnosis (nail unit melanoma), staging, outcomes (overall survival, disease‐free survival, local recurrence, regional recurrence), adverse effects and events including reoperation and readmission data within 30 days could be collected. In terms of procedural data we suggest this should be classified as digit amputation as the control and compared to digit‐sparing surgery. Subcategories could be descriptively presented to include level of amputation and standard versus Mohs surgery for digit‐sparing surgery. Potential confounders include: age, sex, ethnicity, and stage but these could be explored with causal inference methods. Comprehensive analysis of a large and complete data set would enable direct comparison of amputation to digit‐sparing surgery with sufficient follow up and sufficient patient numbers to stratify analysis by tumor stage. This would provide patients and clinicians with robust and cost‐effective information on the treatment of skin cancer of the nail unit.

## AUTHOR CONTRIBUTIONS

**Claire M. Hardie**: Conceptualization; Data curation; Formal analysis; Investigation; Methodology; Project administration; Visualization; Writing—original draft; Writing—review and editing. **Ryckie G. Wade**: Conceptualization; Data curation; Formal analysis; Investigation; Methodology; Writing—original draft; Writing—review and editing. **Justin C. R. Wormald**: Conceptualization; Data curation; Methodology; Writing—review and editing. **Brian Stafford**: Conceptualization; Writing—review & editing. **Faye Elliott**: Methodology; Writing—review and editing. **Julia Newton‐Bishop**: Conceptualization; Supervision; Writing—review and editing. **Donald Dewar**: Conceptualization; Supervision; Writing—review and editing.

## CONFLICT OF INTEREST STATEMENT

Julia Newton‐Bishop: my institution has received a grant from Cancer Research UK to carry out research looking at melanoma survival. Daily fees were paid to me when I was Clinical Lead at the National Institute for Health and Care Excellence (NICE) for the Clinical Melanoma Guideline. I have been reimbursed for travel expenses when asked to give talks at academic meetings. None of these were paid from identifiable commercial entities; conference organizers commonly pool funding sources including commercial companies. I have done legal work for clinical negligence claims in the past and the last case is now coming to an end. These fees were primarily used to support the research group but I did receive one fee from one client personally. My research group is in receipt of a number of research grants from Cancer Research UK, the Medical Research Council, Melanoma Focus, Melanoma Research Alliance and the National Institutes of Health that are for research unrelated to this Cochrane work. A single honorarium was accepted for a talk in 2019; paid to my institution. I receive very small royalties for a textbook published many years ago. I am invited to around three meetings per year where my travel costs are paid by the conference organizers. I have not traveled to meetings where the costs are paid directly by a drug company. I have listed grants and travel expenses paid to enable me to complete my research but I do not believe that they reflect a conflict of interest with respect to this Cochrane work. The other authors declare no conflict of interest.

## Supporting information

Supporting information.

Supporting information.

## Data Availability

Data sharing not applicable—no new data generated.
