# Supplementary material for: Surgical excision methods for skin cancer involving the nail unit: A systematic review
Source: Cochrane Evid Synth Methods. 2023 Oct 16;1(8):e12026. doi: 10.1002/cesm.12026 (PMC11795931; doi:10.1002/cesm.12026)
Supplement: Supplementary file 1 — Supporting information. [file CESM-1-e12026-s001.docx]

Supplementary file 1: Search strategies

**1 Search strategy for the Cochrane Register of Studies (CRSW)**

Top of Form

1. nail or nails or digit or digits or thumb or thumbs or toe or toes or finger or fingers or toenails or toenail or fingernail or fingernails or thumbnail or thumbnails AND INREGISTER
2. subungual or ungual or ungula or periungual or ungueal or unguium AND INREGISTER
3. #1 OR #2
4. carcinoma* or squamous or basal or scc or neoplasm* or melanoma* or cancer* or tumor* or tumour* or malignan* AND INREGISTER
5. excision* or mohs or surgery or surgical or margin* or amputat* or terminali* AND INREGISTER
6. #3 AND #4 AND #5

Bottom of Form

**2 Search strategy for CENTRAL (Cochrane Library)**

Top of Form

#1 MeSH descriptor: [Nail Diseases] explode all trees
#2 MeSH descriptor: [Nails] explode all trees
#3 (digit or digits or thumb or thumbs or toe or toes or finger or fingers or toenail or toenails or fingernail or fingernails or nail or nails or thumbnail or thumbnails):ti,ab,kw
#4 (subungual or ungual or ungula or periungual or ungueal or unguium):ti,ab,kw
#5 {OR #1-#4}
#6 MeSH descriptor: [Carcinoma, Basal Cell] explode all trees
#7 MeSH descriptor: [Carcinoma, Squamous Cell] explode all trees
#8 MeSH descriptor: [Skin Neoplasms] explode all trees
#9 squamous cell carcinoma*:ti,ab,kw
#10 scc:ti,ab,kw
#11 (melanoma* or malignan* or cancer* or neoplasm* or tumor* or tumour* or carcinoma*):ti,ab,kw
#12 {OR #6-#11}
#13 excision*:ti,ab,kw
#14 MeSH descriptor: [Mohs Surgery] explode all trees
#15 MeSH descriptor: [Dermatologic Surgical Procedures] explode all trees
#16 mohs:ti,ab,kw
#17 digit sparing surger*:ti,ab,kw
#18 micrographic surger*:ti,ab,kw
#19 MeSH descriptor: [Margins of Excision] explode all trees
#20 surgical margin*:ti,ab,kw
#21 MeSH descriptor: [Amputation] explode all trees
#22 amputat*:ti,ab,kw
#23 terminali*:ti,ab,kw
#24 {OR #13-#23}
#25 #5 and #12 and #24

Bottom of Form

**3 Search strategy for MEDLINE (Ovid)**

Top of Form

1. exp Nail Diseases/
2. exp Nails/
3. (digit or digits or thumb$ or toe$ or finger$ or toenail$ or fingernail$ or nail$1 or thumbnail$).ti,ab.
4. (subungual or ungual or ungula or periungual or ungueal or unguium).ti,ab.
5. 1 or 2 or 3 or 4
6. exp Carcinoma, Basal Cell/
7. exp Carcinoma, Squamous Cell/
8. exp Skin Neoplasms/
9. squamous cell carcinoma$.ti,ab.
10. scc.ti,ab.
11. (melanoma$ or malignan$ or cancer$ or neoplasm$ or tumor$ or tumour$ or carcinoma$).ti,ab.
12. or/6-11
13. excision$.ti,ab.
14. exp Mohs Surgery/
15. exp dermatologic surgical procedures/
16. mohs.ti,ab.
17. digit sparing surger$.ti,ab.
18. micrographic surger$.ti,ab.
19. exp "margins of excision"/
20. surgical margin$.ti,ab.
21. exp Amputation/
22. amputat$.ti,ab.
23. terminali$.ti,ab.
24. 13 or 14 or 15 or 16 or 17 or 18 or 19 or 20 or 21 or 22 or 23
25. 5 and 12 and 24

Bottom of Form

**4 Search strategy for Embase (Ovid)**

Top of Form

## 1. exp nail disease/ 2. exp nail/ 3. exp thumb/ 4. exp finger/ 5. exp toe/ 6. exp "digit (body part)"/ 7. (digit or digits or thumb$ or toe$ or finger$ or toenail$ or fingernail$ or nail$1 or thumbnail$).ti,ab. 8. (subungual or ungual or ungula or periungual or ungueal or unguium).ti,ab. 9. or/1-8 10. exp squamous cell carcinoma/ 11. exp basal cell carcinoma/ 12. exp skin tumor/ 13. squamous cell carcinoma$.ti,ab. 14. scc.ti,ab. 15. (melanoma$ or malignan$ or cancer$ or neoplasm$ or tumor$ or tumour$ or carcinoma$).ti,ab. 16. exp cutaneous melanoma/ 17. exp skin cancer/ 18. or/10-17 19. exp excision/ 20.  excision$.ti,ab. 21. exp Mohs micrographic surgery/ 22.  exp skin surgery/ 23. mohs.ti,ab. 24. digit sparing surger$.ti,ab. 25.  micrographic surger$.ti,ab. 26. exp surgical margin/ 27. surgical margin$.ti,ab. 28.  amputation/ or finger amputation/ or thumb amputation/ 29. amputat$.ti,ab. 30. terminali$.ti,ab. 31. or/19-30 32. 9 and 18 and 31
