# Supplementary material for: Surgical excision methods for skin cancer involving the nail unit: A systematic review
Source: Cochrane Evid Synth Methods. 2023 Oct 16;1(8):e12026. doi: 10.1002/cesm.12026 (PMC11795931; doi:10.1002/cesm.12026)
Supplement: Supplementary file 2 — Supporting information. [file CESM-1-e12026-s002.docx]

Supplementary file 2: Characteristics of excluded studies

**ACTRN12606000347561**

Wrong patient group

**Azdajic 2018**

Wrong study design

**Campbell 1990**

Wrong study design

**Chakera 2019**

Wrong study design

**Chow 2013**

Wrong study design

**Cohen 2008**

Wrong study design

**Daly 1987**

Wrong study design

**Dika 2012**

Wrong study design

**Dika 2016**

Wrong study design

**Dika 2017**

Wrong study design

**Goettmann 2018**

Wrong study design

**Hudson 1990**

Wrong study design

**Hughes 1985**

Wrong patient population

**Iino 2017**

Wrong study design

**ISRCTN13411879**

Wrong patient population

**Khatri 2016**

Paediatric population

**Klausner 1987**

Wrong intervention

**Mao 2017**

Wrong study design

**Martin 2011**

Wrong study design

**Moehrle 2003**

Wrong study design

**Montagner 2018**

Wrong study design

**Myasnyankin 2016**

Wrong intervention

**Myasnyankin 2017**

Wrong study design

**Park 1992**

Wrong intervention

**Park 2016**

Wrong study design

**Rigby 1992**

Wrong intervention

**Rodenas 2018**

Wrong study design

**Rodriguez-Cuevas 1993**

Wrong intervention

**Shao 1989**

Wrong study design

**Sobjanek 2012**

Wrong study design

**Stanec 2003**

Wrong intervention

**Tan 2007**

Wrong study design

**Tanaka 2019**

Wrong study design

**Taylor 2017**

Wrong study design

**Vasicek 2021**

Wrong study design

**VonRauffer 1978**

Wrong patient population

## References

**ACTRN12606000347561**

ACTRN12606000347561. Multicenter Selective Lymphadenectomy Trial II (MSLT-II). anzctr.org.au/Trial/Registration/TrialReview.aspx?ACTRN=12606000347561 (first received 25 November 2004).

**Azdajic 2018**

Azdajic MD, Lovric I, Franceschi N, Situm M, Vucic M, Buljan M. Early subungual melanoma: A diagnostic and treatment challenge. Dermatologic Therapy 2018;31(6):e12706.

**Campbell 1990**

Campbell WJ, Harper CV, Alderdice JM, Humphreys WG. Subungual melanoma. Irish Journal of Medical Science 1990;159(5):145-6.

**Chakera 2019**

Chakera AH, Quinn MJ, Lo S, Drummond M, Haydu LE, Bond JS, et al. Subunugal melanoma of the hand. Annals of Surgical Oncology 2019;26(4):1035-43.

**Chow 2013**

Chow WT, Bhat W, Magdub S, Orlando A. In situ subungual melanoma: digit salvaging clearance. Journal of Plastic, Reconstructive & Aesthetic Surgery: JPRAS 2013;66(2):274-6.

**Cohen 2008**

Cohen T, Busam KJ, Patel A, Brady MS. Subungual melanoma: management considerations. American Journal of Surgery 2008;195(2):244-8.

**Daly 1987**

Daly JM, Berlin R, Urmacher C. Subungual melanoma: a 25-year review of cases. Journal of Surgical Oncology 1987;35(1):107-12.

**Dika 2012**

Dika E, Piraccini BM, Balestri R, Vaccari S, Misciali C, Patrizi A, et al. Mohs surgery for squamous cell carcinoma of the nail: report of 15 cases. Our experience and a long-term follow-up. British Journal of Dermatology 2012;167(6):1310-4.

**Dika 2016**

Dika E, Patrizi A, Fanti PA, Chessa MA, Reggiani C, Barisani A, et al. The prognosis of nail apparatus melanoma: 20 years of experience from a single institute. Dermatology 2016;232(2):177-84.

**Dika 2017**

Dika E, Piraccini BM, Fanti PA. Management and treatment of nail melanoma. Giornale Italiano di Dermatologia e Venereologia 2017;152(3):197-202.

**Goettmann 2018**

Goettmann S, Moulonguet I, Zaraa I. In situ nail unit melanoma: epidemiological and clinic-pathologic features with conservative treatment and long-term follow-up. Journal of the European Academy of Dermatology and Venereology 2018;32(12):2300-6.

**Hudson 1990**

Hudson DA, Krige JE, Strover RM, King HS. Subungual melanoma of the hand. Journal of Hand Surgery - British Volume 1990;15(3):288-90.

**Hughes 1985**

Hughes LE, Horgan K, Taylor BA, Laidler P. Malignant melanoma of the hand and foot: diagnosis and management. British Journal of Surgery 1985;72(10):811-5.

**Iino 2017**

Iino S, Sato S, Baba N, Maruta N, Takashima W, Oyama N, et al. Two-phase surgery using a dermal regeneration material for nail unit melanoma: Three case reports. Open Dermatology Journal 2017;11:81-6.

**ISRCTN13411879**

ISRCTN13411879. Randomized trial of width of excision of thick cutaneous malignant melanoma. www.isrctn.com/ISRCTN13411879 (first received 19 August 2002).

**Khatri 2016**

Khatri S, Wang M, Andea A, Chan MP. Subungual atypical lentiginous junctional melanocytic proliferation in children and adolescents: A clinicopathologic study. Laboratory Investigation 2016;96:129A.

**Klausner 1987**

Klausner JM, Inbar M, Gutman M. Nail-bed melanoma. Journal of Surgical Oncology 1987;34(3):208-10.

**Mao 2017**

Mao DD, Wen GD, Mu ZL, Cao M, Zhang JZ, Chen X. Squamous cell carcinoma of the nail bed. Chinese Medical Journal 2017;130(7):877-8.

**Martin 2011**

Martin DE, English JC, Goitz RJ. Subungual malignant melanoma. Journal of Hand Surgery - American Volume 2011;36(4):704-7.

**Moehrle 2003**

Moehrle M, Metzger S, Schippert W, Garbe C, Rassner G, Breuninger H. "Functional" surgery in subungual melanoma. Dermatologic Surgery 2003;29(4):366-74.

**Montagner 2018**

Montagner S, Belfort FA, Belda Junior W, Di Chiacchio N. Descriptive survival study of nail melanoma patients treated with functional surgery versus distal amputation. Journal of the American Academy of Dermatology 2018;79(1):147-9.

**Myasnyankin 2016**

Myasnyankin MY, Anisimov VV, Gafton GI, Semiletova YV, Gafton IG. Subungual melanoma. Features of clinic, diagnostics and treatment. Voprosy Onkologii 2016;62(3):474-9.

**Myasnyankin 2017**

Myasnyankin M, Gafton G, Anisimov V, Matsko D, Ivantsov A. Experience of surgical treatment of subungual melanoma. In: Journal of the European Academy of Dermatology and Venereology. Vol. 31 (Supplement 3). 2017:69.

**Park 1992**

Park KGM, Blessing K, Kernohan NM. Surgical aspects of subungual malignant melanomas. Annals of Surgery 1992;216(6):692-5.

**Park 2016**

Park SW, Jang KT, Lee JH, Park JH, Kwon GY, Mun GH, et al. Scattered atypical melanocytes with hyperchromatic nuclei in the nail matrix: diagnostic clue for early subungual melanoma in situ. Journal of Cutaneous Pathology 2016;43(1):41-52.

**Rigby 1992**

Rigby HS, Briggs JC. Subungual melanoma: a clinico-pathological study of 24 cases. British Journal of Plastic Surgery 1992;45(4):275-8.

**Rodenas 2018**

Rodenas JM. Functional surgery, when possible, is the best option for malignant tumors of the nail unit. Actas Dermo-Sifiliograficas 2018;109(8):670.

**Rodriguez-Cuevas 1993**

Rodriguez-Cuevas S, Luna-Perez P. Subungual melanoma. Is elective regional lymph node dissection mandatory? Journal of Experimental and Clinical Cancer Research 1993;12(3):173-8.

**Shao 1989**

Shao YF. Subungual malignant melanoma--30 year review of cases. Zhonghua zhong liu za zhi [Chinese journal of oncology] 1989;11(5):380-2.

**Sobjanek 2012**

Sobjanek M, Michajlowski I, Malek M, Biernat W, Wlodarkiewicz A, Roszkiewicz J. Squamous cell carcinoma of the nail apparatus in the population of Northern Poland. Postepy Dermatologii i Alergologii 2012;29(3):148-51.

**Stanec 2003**

Stanec M, Juzbasic S, Lesar M, Vrdoljak D, Milas I, Nola N, et al. Acral melanoma - A report of 122 cases. Libri Oncologici 2003;31(1-3):61-4.

**Tan 2007**

Tan KB, Moncrieff M, Thompson JF, McCarthy SW, Shaw HM, Quinn MJ, et al. Subungual melanoma: A study of 124 cases highlighting features of early lesions, potential pitfalls in diagnosis, and guidelines for histologic reporting. American Journal of Surgical Pathology 2007;31(12):1902-12.

**Tanaka 2019**

Tanaka K, Nakamura Y, Mizutani T, Shibata T, Tsutsumida A, Fukuda H, et al. Confirmatory trial of non-amputative digit preservation surgery for subungual melanoma: Japan Clinical Oncology Group study (JCOG1602, J-NAIL study protocol). BMC Cancer 2019;19(1):1-6.

**Taylor 2017**

Taylor D, Weiss E. Mohs Micrographic Surgery for Management of Nail Unit Squamous Cell Carcinomas. Dermatologic Surgery 2017;43(10):1302-3.

**Vasicek 2021**

Vasicek B, Erickson T, Lake E. In response to "Descriptive Survival Study of Nail Melanoma Patients Treated With Functional Surgery vs Distal Amputation". Journal of the American Academy of Dermatology 2021;85(6):e351.

**VonRauffer 1978**

Von Rauffer L, Tonak J. Malignant melanoma of the hand: present status of surgical treatment. Chirurgische Praxis 1978;23(3):447-52.
